# Using a two-sample Mendelian randomization design to investigate a possible causal effect of maternal lipid concentrations on offspring birth weight

**DOI:** 10.1093/ije/dyz160

**Published:** 2019-07-23

**Authors:** Liang-Dar Hwang, Deborah A Lawlor, Rachel M Freathy, David M Evans, Nicole M Warrington

**Affiliations:** 1 The University of Queensland Diamantina Institute, The University of Queensland, Brisbane, Australia; 2 Medical Research Council Integrative Epidemiology Unit at the University of Bristol, Bristol, UK; 3 Population Health Sciences, Bristol Medical School, University of Bristol, Bristol, UK; 4 Institute of Biomedical and Clinical Science, College of Medicine and Health, University of Exeter, Exeter, UK; 5 K.G. Jebsen Center for Genetic Epidemiology, Department of Public Health and Nursing, NTNU, Norwegian University of Science and Technology, Norway

**Keywords:** Mendelian randomization, maternal effect, birth weight, high-density lipoprotein cholesterol, low-density lipoprotein cholesterol, triglycerides

## Abstract

**Background:**

The intrauterine environment is critical for fetal growth and development. However, observational associations between maternal gestational lipid concentrations and offspring birth weight (BW) have been inconsistent and ascertaining causality is challenging.

**Methods:**

We used a novel two-sample Mendelian randomization (MR) approach to estimate the causal effect of maternal gestational high-density lipoprotein cholesterol (HDL-C), low-density lipoprotein cholesterol (LDL-C) and triglyceride concentrations on offspring BW. Single nucleotide polymorphisms (SNPs) associated with serum HDL-C, LDL-C and triglyceride concentrations identified in the Global Lipids Genetics Consortium genome-wide association study meta-analysis (*n* = 188 577 European-ancestry individuals; sample 1) were selected as instrumental variables. The effects of these SNPs on offspring BW were estimated using a structural equation model in the UK Biobank and Early Growth Genetics consortium (*n* = 230 069 European-ancestry individuals; sample 2) that enabled partitioning of the genetic associations into maternal- (intrauterine) and fetal-specific effects.

**Results:**

We found no evidence for a causal effect of maternal gestational HDL-C, LDL-C or triglyceride concentrations on offspring BW [standard deviation change in BW per standard deviation higher in HDL-C = −0.005 (95% confidence interval: −0.039, 0.029), LDL-C = 0.014 (−0.017, 0.045), and triglycerides = 0.014 (−0.025, 0.052)].

**Conclusions:**

Our findings suggest that maternal gestational HDL-C, LDL-C and triglyceride concentrations play a limited role in determining offspring BW. However, we cannot comment on the impact of these and other lipid fractions on fetal development more generally. Our study illustrates the power and flexibility of two-sample MR in assessing the causal effect of maternal environmental exposures on offspring outcomes.

**Key Messages**

- We used a novel two-sample Mendelian randomization design to investigate the causal effect of maternal gestational lipid concentrations on offspring birth weight.
- We used summary statistics from genome-wide association studies that predict serum high-density lipoprotein cholesterol, low-density lipoprotein cholesterol, and triglyceride concentrations and birth weight, with the latter being partitioned into maternal- and fetal-specific effects.
- Our findings showed limited support for a causal effect of maternal gestational high-density lipoprotein cholesterol, low-density lipoprotein cholesterol, and triglyceride concentrations on offspring birth weight.
- The same method could be used to examine other maternal exposures putatively causally related to offspring outcomes assessed later in life.

## Introduction

On average, babies born at term below the 25th percentile or above the 85th percentile of the population distribution for birth weight (BW) in high-income countries are at higher risk of adverse perinatal outcomes, such as stillbirth, neonatal mortality, respiratory problems and the need for admission to neonatal intensive care.^1^ Additionally, babies born of lower and higher BW are at increased risk of adverse future health. For example, inverse associations have been identified between BW and coronary heart disease,^2^ positive associations with body mass index (BMI)^3^ and most cancers^4^ and U-shaped associations with future diabetes risk.^5^ Understanding which maternal traits during pregnancy causally affect offspring BW will assist in understanding the mechanisms underlying these associations and defining a healthy BW range.

Maternal serum lipid concentrations are one such trait that could influence offspring BW. Observational studies have reported inconsistent results regarding the relationship between maternal lipid concentrations and offspring BW. For example, maternal serum high-density lipoprotein cholesterol (HDL-C) concentrations have been reported to have positive,^6^ negative,^7–9^ and no^10–12^ association with offspring BW. In the case of maternal low-density lipoprotein cholesterol (LDL-C), most studies have reported no association with offspring BW^7–10^ although there is some evidence for a negative association between smaller subclasses of LDL-C and offspring BW.^10^ Higher maternal triglyceride concentrations have been shown to be associated with higher offspring BW;^6^^,^^11^^,^^12^ however, some associations were only observed when the study samples were stratified by maternal pre-pregnancy BMI (i.e. in mothers with pre-pregnancy BMI between 18.5 and 24.9 kg/m^2 ^^8^,^9^ or overweight/obese mothers with BMI >25 kg/m^2^).^7^ Bivariate linkage disequilibrium (LD) score regression analyses^13^^,^^14^ applied to a large-scale genome-wide association study (GWAS) meta-analysis of BW has shown negative genetic correlations of it with LDL-C and triglyceride concentrations, and a positive genetic correlation with HDL-C.^15^ However, it is unclear whether these genetic correlations reflect pleiotropic effects of genetic loci influencing both traits or are a consequence of maternal blood lipids causally affecting offspring BW.

Mendelian randomization (MR) is a method commonly used in epidemiology to estimate the causal relationship between a modifiable environmental exposure of interest and a medically relevant trait or disease.^16^ It uses genetic variants, most commonly single nucleotide polymorphisms (SNPs), robustly associated with the modifiable environmental exposure of interest as instrumental variables to estimate the causal effect. In the context of this manuscript, the environmental exposures of primary interest are maternal lipid concentrations during pregnancy (i.e. maternal HDL-C, maternal LDL-C and maternal triglycerides) and the medically relevant outcome is offspring BW.

**Figure 1.**
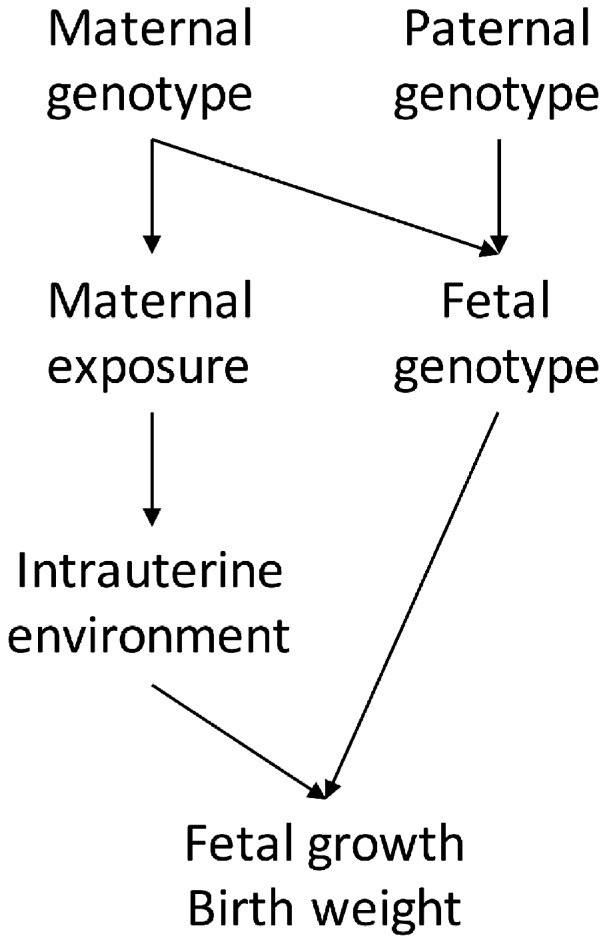
A directed acyclic graph (DAG) showing maternal and fetal genetic effects on offspring birth weight. Offspring birth weight can be influenced by both maternal and fetal genotypes, the former operating through the intrauterine environment and via fetal genotype. As fetal genotype is determined by maternal and paternal genotypes, to accurately estimate the association between maternal genotype and offspring birth weight we need to partition the overall genetic effect into maternal- and fetal-specific genetic components. This DAG shows the key pathways and assumptions of our analyses. We have not depicted the (unknown) paths through which fetal genotype would influence fetal growth and birth weight. Note, also that we are assuming that phenotypes relating to fetal genotype do not affect the intrauterine environment, whereas it is known that waste products form the fetus are transferred back across the placenta into the maternal circulation. If fetal genotype influences the production or transfer of waste products, this assumption will be violated.

Unlike MR studies investigating the causal relationship between two adult traits, estimating the causal effect of maternal exposures on offspring outcomes using MR is difficult due to the fact that maternal (and paternal) genotype determine offspring genotype. Specifically, any association between maternal genotype and offspring outcome could be mediated by the child’s genotype rather than through the maternal genotype operating on the intrauterine environment (Figure 1). To ensure an unbiased causal estimate of the maternal environmental exposure on offspring outcome, one could perform MR analyses in genotyped mother–offspring duos and statistically correct for offspring’s genotype by conditioning on it.^6^^,^^17^ However, there are only a small number of cohorts worldwide that have genotyped mother–offspring duos, meaning that such analyses may be underpowered, especially in comparison to two-sample MR studies where different but maximized samples of individuals report SNP–exposure and SNP–outcome associations. Our previous MR study used genotyped mother–offspring duos in an attempt to examine the effect of maternal obesity related traits, including HDL-C and triglyceride concentrations, on offspring BW.^6^ Results suggested a possible causal inverse effect of maternal triglycerides on BW (i.e. opposite direction to most previous multivariable regression results). However, despite bringing together as many studies with genotyped maternal–offspring duos as possible in a sample size of 11 031 the effect was imprecisely estimated with wide confidence intervals: difference in mean BW per 1-standard deviation (SD) higher triglycerides = −33 g [95% confidence intervals (CIs): −86, 20].^6^ For HDL-C the results were more clearly null [−1 g (−55, 54); *n* = 9176].

**Figure 2.**
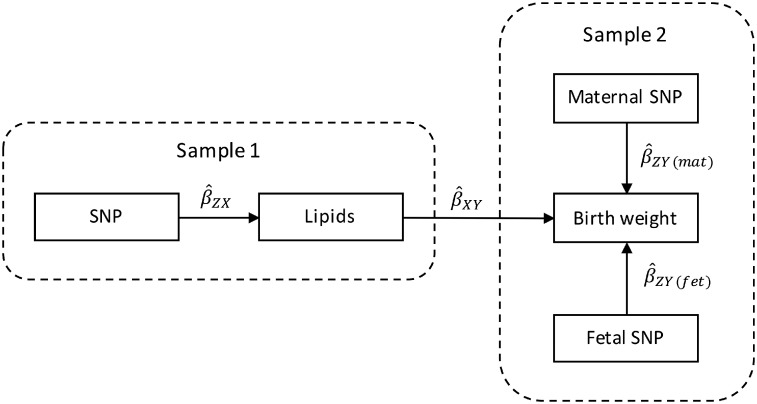
Two-sample Mendelian randomization testing the causal effect of maternal and fetal serum lipid concentrations on offspring birth weight. Estimates of the SNP-lipid association (${\hat{\beta }}_{\mathrm{ZX}}$) are calculated in sample 1. The association between these same SNPs and birth weight (BW) is then estimated in sample 2, except in this case the genetic association is partitioned into maternal (${\hat{\beta }}_{\mathrm{ZY}(\mathrm{mat})}$) and fetal (${\hat{\beta }}_{\mathrm{ZY}(\mathrm{fet})}$) effects on BW. These estimates are combined to yield estimates for each SNP of the maternal effect of serum lipid concentrations on offspring BW (${\hat{\beta }}_{\mathrm{XY}(\mathrm{mat})}=\frac{{\hat{\beta }}_{\mathrm{ZY}(\mathrm{mat})}}{{\hat{\beta }}_{\mathrm{ZX}}}$), and the offspring effect of their own serum lipid concentrations on their own BW (${\hat{\beta }}_{\mathrm{XY}(\mathrm{fet})}=\frac{{\hat{\beta }}_{\mathrm{ZY}(\mathrm{fet})}}{{\hat{\beta }}_{\mathrm{ZX}}}\;$). Finally, the estimates at each SNP are combined using the two-sample MR approaches, including inverse-variance weighted analysis (${\hat{\beta }}_{\mathrm{IVW}}$) and the various sensitivity analyses, to produce an overall causal estimate of serum lipids on BW.

An alternative solution, which we adopt, is to employ two-sample MR to estimate the causal effect of maternal lipid concentrations on offspring BW, but using unbiased estimates of the maternal genetic effect on BW from a very large GWAS meta-analysis of the UK Biobank and the Early Growth Genetics (EGG) Consortium.^18^ Recently, we showed how structural equation modelling (SEM) could be used to partition genetic effects on BW into maternal-specific and fetal-specific components in the UK Biobank Study, even in the absence of genotyped mother–offspring duos.^19^ Our design relies on the fact that genotyped individuals in the UK Biobank reported their own BW, and (in the case of women) the BW of their first child. The SEM can also include summary statistics from the EGG consortium GWAS of own or offspring BW, increasing the sample size and therefore the precision of the maternal- and fetal-specific genetic effect estimates.^19^ Maternal genetic effects estimated using this study design represent the association between maternal genotype and offspring BW with the effect of child’s genotype removed (i.e. similar to an analysis where child’s genotype has been included as a covariate in a study of genotyped mother–offspring duos, but in vastly larger samples of individuals). Using a two-sample MR framework, these estimates of the maternal genetic effect on BW can subsequently be combined with SNP-exposure estimates for the maternal exposures we are interested in, allowing us to maximize sample size and statistical power (Figure 2).

In a recent study using this novel two-sample MR approach, we demonstrated a positive causal effect of maternal height and fasting glucose and inverse causal effect of blood pressure on offspring BW^18^. In the present study we used this two-sample MR design to investigate a possible causal effect of maternal lipid concentrations on offspring BW. We performed two-sample inverse variance weighted MR, and also a series of sensitivity analyses including MR Egger regression,^20^ weighted median^21^ and mode-based^22^ analyses, with SNP-lipid associations from the Global Lipids Genetics Consortium^23^ and the maternal SNP-BW associations from the UK Biobank and the EGG Consortium.^18^

## Methods

### Two-sample inverse variance weighted Mendelian randomization analysis

To investigate the causal relationship between maternal lipid concentrations and offspring BW we initially performed two-sample inverse variance weighted (IVW) MR analyses^24^^,^^25^ using GWAS summary results data from the Global Lipids Genetics Consortium (*n* = 188 577 European-ancestry individuals; sample 1)^23^ and the summary results data from the GWAS meta-analysis of BW in the UK Biobank and the EGG Consortium (*n* = 230 069 European-ancestry individuals; sample 2).^18^ The magnitude of the causal effect (${\hat{\beta }}_{\mathrm{IVW}}$) was estimated as the average of the SNP-outcome effect ${(\hat{\beta }}_{\mathrm{ZY}(\mathrm{mat})})$ divided by the SNP-exposure effect $\left({\hat{\beta }}_{\mathrm{ZX}}\right)$ for all SNPs associated with an exposure, and weighted by the inverse variance of the SNP-outcome effect ${(\hat{\beta }}_{\mathrm{ZY}(\mathrm{mat})})$. The regression slope (ratio) in IVW analyses is forced through a zero intercept (i.e. assuming that there is no unbalanced horizontal pleiotropy and hence a zero difference in exposure results in zero difference in BW).

The exposure variables of interest were maternal concentrations of three serum lipid fractions: HDL-C, LDL-C and triglycerides. A total of 185 independent SNPs have previously been robustly associated with these three lipid fractions (*P* < 5.0 x 10^–8^) and were selected as instrumental variables (IVs) for MR analyses (Supplementary Table 1, available as Supplementary data at *IJE* online). Of these SNPs, 96 were associated with HDL-C, 82 were associated with LDL-C, and 60 were associated with triglycerides, all at the genome-wide significance level of *P* < 5.0 x 10^–8^. We refer to these SNPs as the ‘Unrestricted Set’ of SNPs for each of the three serum lipids. As many of these SNPs were associated with more than one lipid fraction, which potentially violates the exclusion restriction assumption of IVW MR analysis, we created a ‘Restricted Set’ of SNPs, which had strong evidence of association with one lipid fraction only (i.e. *P* < 5.0 x 10^–8^ for one lipid and *P* > 0.05 for the other two). This reduced the number of associated SNPs to 15 SNPs for HDL-C, 20 SNPs for LDL-C, and 3 SNPs for triglycerides (Supplementary Table 1, available as Supplementary data at *IJE* online). We did not perform MR analyses using the ‘Restricted Set’ for triglycerides because the 3 SNPs together only accounted for <0.1% of the phenotypic variance in serum triglyceride concentrations and calculations indicated low power to detect a causal relationship of maternal triglycerides on BW.

The outcome of interest was offspring BW. Because of concerns regarding confounding due to the effect of maternal genotypes on offspring genotypes, in our MR analyses we used maternal-specific genetic effects on offspring BW estimated using the SEM for each of the lipid-associated SNPs, as illustrated in Figure 2. The maternal SNP effects on BW estimated using the SEM are equivalent to the regression of offspring BW on maternal genotype conditional on offspring genotype. The maternal- (and fetal-) specific effects on offspring BW for each of the genetic instruments are presented in Supplementary Table 1, available as Supplementary data at *IJE* online. We note that the SNP effects on BW were obtained from a GWAS meta-analysis^18^ and that BW was estimated in a variety of different ways including by retrospective self-report in the UK Biobank cohort. Where possible, studies in the EGG consortium were adjusted for gestational age prior to GWAS analysis; as gestational age is not recorded in UK Biobank this adjustment was not possible.

To investigate the possibility of directional pleiotropy in our data, we used funnel plots^20^ that plot instrument strength against the causal estimates for all IVs, and sensitivity analyses (see below) that relax some of the horizontal pleiotropy assumptions. Asymmetry in funnel plots suggests that causal estimates from weaker variants tend to be skewed in one direction and may indicate directional horizontal pleiotropy.

### Sensitivity analyses

As the IVW approach could yield biased estimates in the presence of horizontal pleiotropy (i.e. where some SNPs influence the outcome via additional paths other than via the exposure), we performed four sensitivity analyses using additional MR models, MR Egger regression,^20^ the weighted median estimator,^21^ and the simple and weighted mode-based estimator.^22^

MR Egger regression involves a weighted linear regression of ${\hat{\beta }}_{\mathrm{ZY}(\mathrm{Mat})}$ on ${\hat{\beta }}_{\mathrm{ZX}}$ where the intercept from the regression is free to vary. The slope of the regression provides an estimate of the causal effect of the exposure on the outcome that is free from directional horizontal pleiotropy.^20^ The presence of an intercept that is different from zero indicates overall directional pleiotropy, which would bias the IVW estimate. As the assessment of directional pleiotropy using MR Egger can be affected by the orientation of the IVs,^26^ we oriented the effect alleles of all IVs to have positive effects on the exposure prior to all MR Egger analyses. The validity of MR Egger regression relies on an assumption known as the ‘InSIDE (INstrument Strength Independent of Direct Effect) assumption’, which states that the pleiotropic effects of SNPs must be independent of their strength as instruments. In other words, the strength of association between the SNPs and the outcome via pathways other than through the exposure must be independent of the strength of the SNP-exposure associations. This is a weaker assumption than requiring the complete absence of horizontal pleiotropy to obtain valid causal estimates, which is the assumption required in traditional IVW MR analysis. However, although MR Egger regression provides a more robust estimate of the causal effect of the exposure on the outcome, its power to detect a causal effect is much lower than IVW analyses.^20^ MR Egger regression also makes the assumption that the SNP-exposure associations are estimated without error. To check whether this ‘NOME (NO Measurement Error) assumption’ was violated in the two-sample MR Egger regression context, we used the $I_{\text{GX}}^{2}$ statistic^27^ to estimate the potential relative (dilution) bias due to measurement error. The I2_GX_ statistic is a value ranging between 0 and 1, with the value close to 1 indicating that the SNP-exposure associations are sufficiently heterogeneous and the uncertainty is relatively small compared with its variability so the violation of NOME is negligible.

The weighted median estimator approach uses the weighted median of the ratios (i.e. the ratio of ${\hat{\beta }}_{\mathrm{ZY}(\mathrm{Mat})}$ and ${\hat{\beta }}_{\mathrm{ZX}}$) of all the IVs, as opposed to the weighted mean that is used in the IVW approach. The approach provides an asymptotically consistent estimate of the causal effect and tolerates up to (but not including) 50% of the weight coming from invalid IVs (e.g. IVs that violate the exclusion restriction assumption).^21^

The simple mode-based estimator clusters the SNPs into groups based on similarity of causal effect estimates.^22^ The causal effect is estimated based on the cluster that has the largest number of SNPs. The weighted mode-based estimator weights the number of SNPs within each cluster by the inverse variance of each SNP’s effect on the outcome and returns the casual estimate based on the cluster that has the largest weighted number of SNPs. The mode-based methods return an unbiased estimate of the causal effect if the SNPs within the cluster with the largest number of SNPs (or the largest weighted number for the weighted model) are all valid instruments, which is also referred as the ‘ZEMPA assumption (ZEro Model Pleiotropy Assumption)’.

If the five MR models (IVW, MR Egger regression, weighted median estimator, simple and weighted mode-based estimator), which make different assumptions regarding instrument validity, produce similar estimates of the causal effect, then we can be more confident in the robustness of our findings.

### Multivariable Mendelian randomization analysis

Two multivariable MR analyses, multivariable IVW regression^28^ and multivariable MR Egger regression,^29^ were performed as additional sensitivity analyses. These multivariable approaches take into account the correlation between the three lipid fractions and the fact that the SNPs used in the MR analyses using the ‘unrestricted’ SNP set are often associated with more than one lipid fraction (i.e. 147 and 44 SNPs in our analyses were associated with at least two lipid fractions with *P* < 0.05 and 5.0 x 10^–8^, respectively; Supplementary Table 1, available as Supplementary data at *IJE* online).

The multivariable IVW regression was performed by regressing ${\hat{\beta }}_{\mathrm{ZY}(\mathrm{Mat})}$ on ${\hat{\beta }}_{\mathrm{ZX}}$ for all three lipid fractions (i.e. three sets of$\;{\hat{\beta }}_{\mathrm{ZX}}$ across all 185 SNPs) in a single regression model. Inverse variance weights of the ${\hat{\beta }}_{\mathrm{ZY}(\mathrm{Mat})}$ were used in the regression model and the intercept was initially fixed at zero. The multivariable IVW regression analyses were performed using all 185 SNPs at once with the causal effect for each exposure estimated from the regression coefficients for the corresponding set of ${\hat{\beta }}_{\mathrm{ZX}}$. A critical assumption, additional to the univariate assumptions, for multivariable MR is that the relationship between genetic instruments and the outcome is only mediated by the exposures considered in the analysis.^28^

The multivariable MR Egger regression was performed also by including all 185 lipid-associated SNPs (i.e. three sets of ${\hat{\beta }}_{\mathrm{ZX}}$) in a single regression model. Similar to the univariate MR Egger regression model, the multivariable model allows the intercept to vary, with a non-zero estimate of the intercept suggestive of horizontal pleiotropy. We performed three separate analyses. In each of these analyses the SNP alleles were oriented to reflect the direction of association at a single lipid fraction (i.e. first HDL-C, then LDL-C and then triglycerides) to estimate its causal effect. The advantage of the multivariable MR Egger regression model over the univariate one is that it reduces the bias when the amount of residual effect on the outcome (i.e. SNP-outcome associations not via the exposure) is decreased by including additional exposures.

We used the TwoSampleMR package^30^ (version 0.4.11) in R (version 3.4.3) to perform IVW, MR Egger regression, weighted median, mode-based estimator and multivariable IVW MR analyses. We used the codes provided on https://mrcieu.github.io/TwoSampleMR/ to run the analyses. We used the R scripts provided in a methods paper^30^ to perform the multivariable Egger regression analysis.

## Results

**Figure 3.**
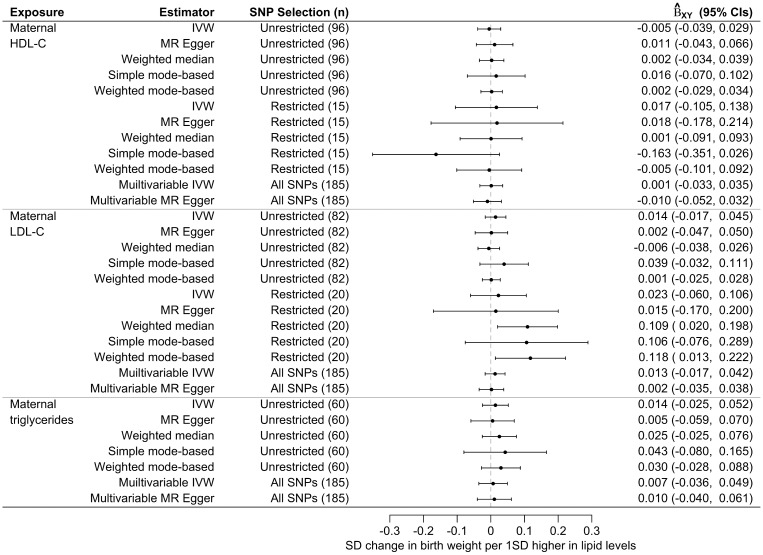
Mendelian randomization estimates of the causal effect of maternal serum high-density lipoprotein cholesterol (HDL-C), low-density lipoprotein (LDL-C), and triglyceride concentrations on offspring birth weight. Causal effects are estimated using five univariate MR models [i.e. inverse variance weighted (IVW), MR Egger regression, weighted median, simple mode-based, and weighted mode-based] and two multivariable MR models (i.e. multivariable IVW regression and multivariable MR Egger regression). Each univariate MR model was fitted twice using an ‘Unrestricted Set’ of SNPs (i.e. all SNPs strongly associated with the exposure with *P* < 5.0 x 10^–8^) and a ‘Restricted Set’ of SNPs (i.e. SNPs strongly associated with the exposure with *P* < 5.0 x 10^–8^ but not associated with the other two lipids with *P* > 0.05). Forest plots show the estimated causal change (${\hat{\beta }}_{\mathrm{XY}}$) in standard deviations (SD) of offspring birth weight per 1SD higher in serum level of HDL-C, LDL-C or triglycerides with 95% confidence intervals (CIs).

There was little evidence for a causal effect of maternal gestational concentrations of HDL-C, LDL-C or triglycerides on offspring BW (Figure 3). Each estimate represents the estimated causal difference in mean SD of offspring BW per 1SD higher maternal concentration of HDL-C, LDL-C or triglycerides. We found no evidence of directional horizontal pleiotropy at any of the IVs for the three lipid fractions; the estimates of the intercept of both the univariate and multivariable MR Egger regressions were approximately zero in all analyses (Supplementary Table 2, available as Supplementary data at *IJE* online) and there was a lack of asymmetry of the funnel plots (Supplementary Figure 1, available as Supplementary data at *IJE* online). We also found little evidence for violation of the ‘NOME assumption’ for the MR Egger regression as the I^2^_GX_ statistics were >97.5% for all analyses (univariate and multivariable MR regressions for all three lipids; Supplementary Table 2, available as Supplementary data at *IJE* online).

### Maternal serum concentrations of HDL-C and offspring birth weight

**Figure 4.**
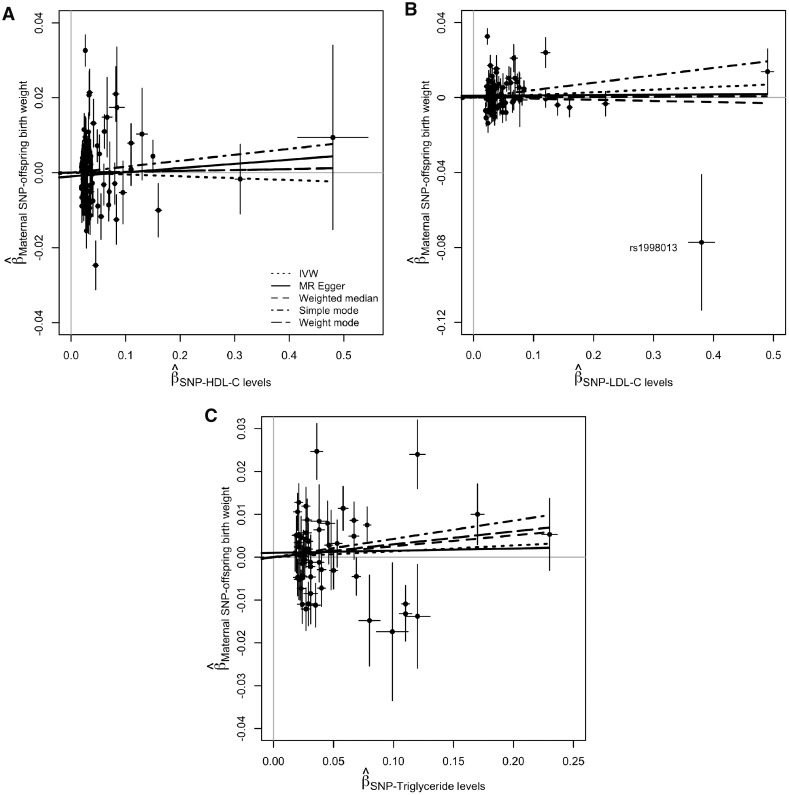
Scatter plots of the estimated SNP effects on offspring birth weight plotted against the estimated SNPs effects on the maternal (A) HDL-C, (B) LDL-C, and (C) triglyceride concentrations The slopes of the lines are the estimated causal effects of the maternal lipid concentrations on offspring birth weight, estimated using different MR methods (i.e. inverse variance weighted, MR Egger regression, weighted median, simple mode-based, and weighted mode-based). An outlier SNP, rs1998013, is labelled in (B). It is distal from all other LDL-C associated SNPs and drives the causal effect toward the null.

The two-sample MR IVW analysis showed no causal effect of maternal HDL-C level and offspring weight [SD change in BW per 1SD higher in HDL-C = −0.005 (−0.039, 0.029)]. This was supported by all univariate and multivariable sensitivity analyses (Figures 3 and 4A), and regardless of whether SNPs from the ‘Unrestricted Set’ or the ‘Restricted Set’ were used in the analysis.

### Maternal serum concentrations of LDL-C and offspring birth weight

While no causal effect was found in the univariate IVW analysis [SD change in BW per 1SD higher in LDL-C = 0.014 (−0.017, 0.045)], a higher genetically predicted level of maternal LDL-C tended to be associated with a higher offspring BW in the weighted median [SD change in BW per 1SD higher in LDL-C = 0.109 (0.020, 0.198)] and weighted mode-based [SD change in BW per 1SD higher in LDL-C = 0.118 (0.013, 0.222)] analyses using the ‘Restricted Set’ of SNPs (Figures 3 and Supplementary Figure 2, available as Supplementary data at *IJE* online). However, this estimated positive causal effect was not supported by all other univariate and multivariable analyses. The SNP rs1998013 appears to be an outlier in the scatterplot (Figure 4B). It is distal from all other LDL-C associated SNPs and it has the largest absolute effect on BW and the second largest effect on LDL-C that would drive the causal effect toward the null, which is against the overall effect of all other SNPs. We excluded it and re-ran the unrestricted and restricted analyses; the results remained unchanged (Supplementary Figure 3 and Supplementary Table 3, available as Supplementary data at *IJE* online).

### Maternal serum concentrations of triglycerides and offspring birth weight

Similar to the previous two lipid fractions, no causal effect of maternal triglycerides on offspring BW was detected in the univariate IVW analysis [SD change in BW per 1SD higher in triglycerides = 0.014 (−0.025, 0.052)], which was supported by the sensitivity analyses (Figures 3 and 4C).

## Discussion

We employed a novel methodological approach to assess the causal effect of maternal concentrations of LDL-C, HDL-C and triglycerides on offspring BW using a two-sample MR framework. The major difference between our approach and standard two-sample MR is that the SNP-outcome associations (i.e. SNP-BW associations) have been partitioned into maternal and fetal genetic components, which has allowed us to estimate the effect of maternal exposures on BW, independent of the fetal genetic effects. We applied this method to examine the causal effect of maternal gestational LDL-C, HDL-C and triglycerides on offspring BW, while controlling for fetal genotype, and found little support for a causal effect of these on offspring BW.

Results from this study are consistent with our previous MR study using a smaller sample of genotyped mother–offspring pairs for HDL-C although we do not find any evidence of a suggested inverse effect of triglycerides on birthweight reported in that previous study.^6^ In that study, polygenic scores of HDL-C and triglyceride concentrations were calculated using GWAS summary data on lipid concentrations^31^ and the maternal genotypes, and no association was found between genetic scores and offspring BW after conditioning on offspring genotype, although a suggested effect of triglycerides was noted (albeit with very wide confidence intervals). The present study has extended this earlier work by using a much larger sample of individuals and using a range of sensitivity analyses to investigate possible bias through genetic pleiotropy. We were also able to explore effects of LDL-C which was not done in that previous study. As an indication of the power of the novel two-sample MR approach used here we can compare the results for the estimated effect of triglycerides on BW from our two studies. Assuming that the SD for BW is 454 g (the mean value in the EGG consortium) our result for an effect of maternal triglycerides on BW is 6.36 g (95%CI: −11.35, 23.61). Compared with our previous study result [−33 g (95%CI: −86, 20)] we now have a more precise estimate suggesting no evidence of an important effect.

We emphasize that the null associations we found do not necessarily mean that maternal LDL-C, HDL-C and triglyceride concentrations have no effect on the developing fetus. They suggest that these maternal circulating lipid fraction concentrations have no effect on the overall growth of the fetus that is reflected by BW. Maternal circulating LDL-C, HDL-C and triglycerides could still impact the development of a particular organ, growth during a particular period of pregnancy, or differential growth of fat, lean and skeletal tissue, but we could not detect these effects by looking only at BW. Several small detailed studies have documented how maternal lipid and fatty acid concentrations change across pregnancy, showing particularly marked increases in triglycerides and more modest increases in cholesterols and phospholipid concentrations together with an increase in the proportion of triglycerides carried in very low-density and low-density lipoprotein particles towards the end of pregnancy.^32^ More recently larger epidemiological studies have shown marked changes in lipids and lipoproteins as women become pregnant, which then return to normal after pregnancy.^33^ Just how different lipid fractions and fatty acids are carried across the placenta and are used by the fetus for healthy growth and development is unclear, but with the emergence of genome-wide analyses of a much wider range of lipids and fatty acids^34^^,^^35^ it will be possible to extend the methods used in this study to determine the impact of more refined measures of maternal gestational lipid metabolism on BW. Additionally, BW was obtained from offspring born at full term (>37 weeks of gestation), so we are unable to comment on whether these maternal lipid fraction concentrations have an effect on the BW of pre-term offspring.

A key assumption of our study design is that SNPs associated with lipid concentrations in the general population of men and (non-pregnant) women are appropriate instruments for lipid concentrations in mothers during pregnancy. Our concern arises from the fact that maternal lipid concentrations change dramatically during pregnancy,^36^^,^^37^ there may be sex differences in the magnitude of association between SNPs and lipid concentrations, and that lipid concentrations measured at different gestational stages may have different observational associations with BW.^12^ We previously showed that maternal gestational HDL-C and triglycerides were strongly correlated with polygenic risk scores constructed from HDL-C and triglyceride associated SNPs in the general population (although LDL-C was not examined), suggesting that the same genetic variants proxy lipid concentrations in pregnant and non-pregnant individuals (see eTable 5 of Tyrrell *et al*.).^6^ Larger studies investigating the effect of the lipid-associated SNPs in pregnant women are required.

Another potential complication in interpreting the results of these sorts of MR analyses is the extent to which one can be sure that the causal estimates reflect the effect of the prenatal environment on the developing offspring. For example, it is possible that variation in circulating lipid concentrations before conception might affect oocyte quality and then have downstream effects on offspring BW.^17^^,^^38^ It is also possible that the causal estimates could reflect the effect of postnatal influences on offspring phenotype. Although this is unlikely to be a concern for perinatal traits like BW where post-natal effects have not had time to exert appreciable effects on the offspring phenotype, it is a potential concern for later life phenotypes, particularly if the investigator is specifically trying to ascribe a causal role for the prenatal environment. One way to increase confidence that the MR results do indeed reflect causal effects of the prenatal environment would be to perform similar analyses using paternal genotype (conditional on offspring genotype). A significant causal relationship would suggest that the post-natal environment is also likely to be important in determining the offspring phenotype, though such a comparison, and indeed our results presented here, assume that the fetal genotype does not have an effect on the intrauterine environmental exposure. Fetal waste products cross the placenta into the maternal circulation and it is possible that fetal genotypes (that are determined by maternal genotypes for the intrauterine exposure of interest) might influence the secretion of fetal waste products into the maternal circulation, which in turn might influence the exposures of interest. Our method, like two-sample MR in general, is statistically inefficient for exploring non-linear effects or stratified effects (e.g. by maternal BMI).

There are no obvious technical reasons why our method cannot be applied more generally to outcomes other than BW. The major practical limitation, however, is the availability of large-scale cohorts with maternal genotypes along with both maternal and offspring phenotypes so that unbiased estimates of maternal genetic effects on offspring outcomes can be made available to researchers for two-sample MR studies. One way to directly facilitate the availability of these kinds of data to the scientific community would be for researchers to publish summary GWAS results of maternal genotype and offspring phenotype, conditional on offspring genotype. However, performing conditional association analyses across the genome is computationally intensive, and may be difficult for many researchers to implement. A more practical alternative might be for investigators to deposit unconditional GWAS results of the association between maternal genotype and offspring phenotype and separate unconditional GWAS results between offspring genotype and offspring phenotype. These summary results could then be used in a structural equation model to generate unbiased estimates of maternal and fetal genetic effects on offspring phenotype, even when the degree of sample overlap is unknown.^19^ Appropriate standard errors could be obtained in these models by estimating the degree of sample overlap using bivariate LD score regression^13^ and weighting the likelihood appropriately. It is likely that similar results could also be obtained by using other methods for conditional GWAS.^39–41^

## Conclusion

Maternal gestational LDL-C, HDL-C and triglyceride concentrations do not appear to play a causal role in determining offspring BW. Our study illustrates how causal estimates of maternal environmental exposures on offspring BW can be generated in extremely large samples of individuals using a two-sample MR framework, obviating the requirement for mother–offspring duos with individual level genotypes. The implication is that the same method could be used to examine other maternal exposures putatively causally related to offspring BW, and potentially the relationship between maternal exposures and other offspring outcomes once large scale GWAS of maternal effects are publicly available.

## Funding

D.M.E. is supported by an NHMRC Senior Research Fellowship (GNT1137714) and this work was supported by a project grant (GNT1125200). D.A.L’s contribution to this piece is supported by the European Research Council under the European Union's Seventh Framework Programme (FP/2007–2013) / ERC Grant Agreement (Grant number 669545; DevelopObese), the European Union’s Horizon 2020 research and innovation programme under grant agreement No 733206 (LifeCycle), the United States National Institutes of Health (NIH): National Institute of Diabetes and Digestive and Kidney Diseases (R01 DK10324), and the British Heart Foundation (AA/18/7/34219). D.M.E and D.A.L work in a UK Medical Research Council (MRC) Unit that is supported by the University of Bristol and MRC (MC_UU_00011/6), and D.A.L. is a National Institute of Health Research Senior Investigator (NF-SI-0611–10196). N.M.W. is supported by an Australian National Health and Medical Research Council Early Career Fellowship (GNT1104818). R.M.F. is supported by Sir Henry Dale Fellowship (Wellcome Trust and Royal Society grant: WT104150). This research has been conducted using the UK Biobank Resource (Reference 12703). Access to the UKBB study data was funded by University of Queensland Early Career Researcher Grant (2014002959). L.-D.H is supported by a National Health and Medical Research Council project grant (GNT1125200). This research was carried out at the Translational Research Institute, Woolloongabba, QLD 4102, Australia. The Translational Research Institute is supported by a grant from the Australian Government. No funders influence the study design, data collection or interpretation of results. The views expressed in this paper are those of the authors and not necessarily any funder.

**Conflict of** **interest**: D.A.L has received research support from several national and international Government and charity funders, and from Medtronic Ltd and Roche Diagnostics in the last 10-years. All other authors report no conflicts of interest.

## Supplementary Material

dyz160_Supplementary_Data
