# Supplementary material for: Using a two-sample Mendelian randomization design to investigate a possible causal effect of maternal lipid concentrations on offspring birth weight
Source: Int J Epidemiol. 2019 Jul 23;48(5):1457–67. doi: 10.1093/ije/dyz160 (PMC6857765; doi:10.1093/ije/dyz160)
Supplement: dyz160_Supplementary_Data [file dyz160_supplementary_data.zip › dyz160-Suppl_data/Supplementary_Data1.docx]

**Using a two-sample Mendelian randomization design to investigate a possible causal effect of maternal lipid concentrations on offspring birth weight**

Liang-Dar Hwang^1^, Deborah A. Lawlor^2,3^, Rachel M. Freathy^4^, David M. Evans^1,2,3*^, Nicole M. Warrington^1,5*^

^1^The University of Queensland Diamantina Institute, The University of Queensland, Brisbane, Australia

^2^Medical Research Council Integrative Epidemiology Unit, University of Bristol, Bristol, UK

^3^Population Health Sciences, Bristol Medical School, University of Bristol, Bristol, UK

^4^Institute of Biomedical and Clinical Science, College of Medicine and Health, University of Exeter, Exeter, UK

^5^K.G. Jebsen Center for Genetic Epidemiology, Department of Public Health and Nursing, NTNU, Norwegian University of Science and Technology, Norway

*Joint senior authors

Corresponding author: Nicole M. Warrington

Email: n.warrington@uq.edu.au

Telephone: +61 7 3443 7347

Address: University of Queensland Diamantina Institute

Level 7, 37 Kent St

Translational Research Institute

Woolloongabba, QLD 4102

Australia


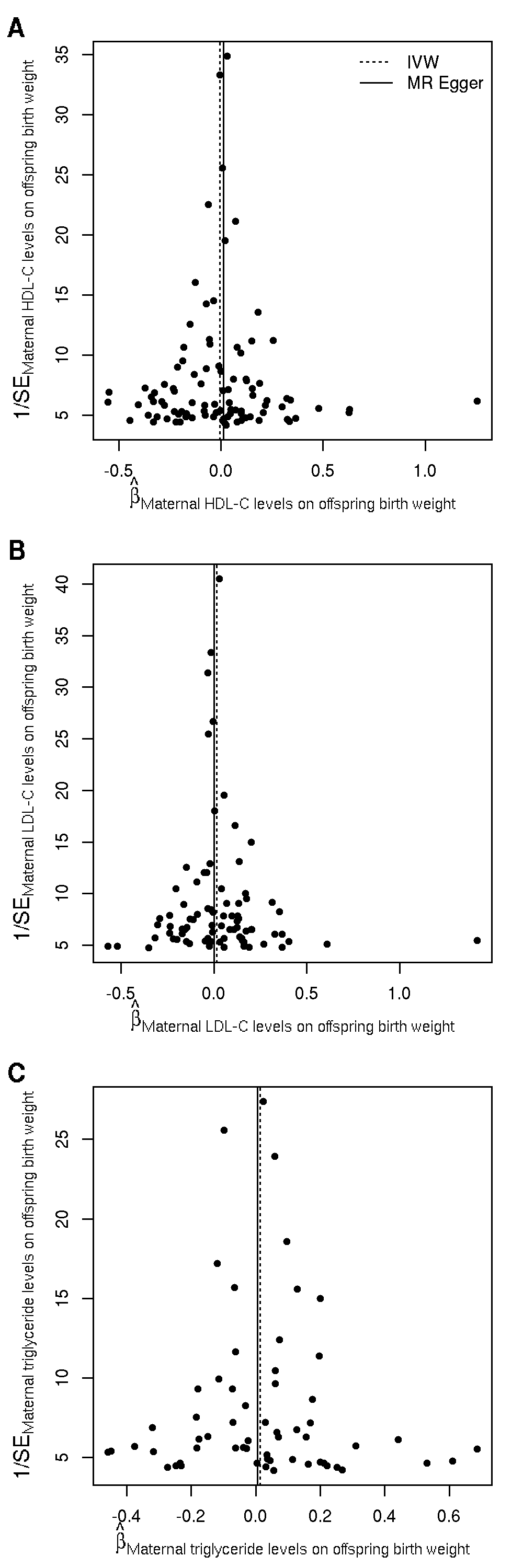
**Supplementary Figure 1. Funnel plots of instrument strength (y-axis) plotted against effect size of causal estimates (x-axis).** Lack of asymmetry in the funnel plot for each of (A) HDL-C, (B) LDL-C, and (C) triglycerides suggests no overall directional horizontal pleiotropy (n = 96, 82, and 60 SNPs for HDL-C, LDL-C, and triglycerides respectively) on offspring birth weight. Vertical lines show the causal estimates using all SNPs associated with the corresponding lipid combined into a single instrument for MR IVW and MR Egger regression analyses.

**
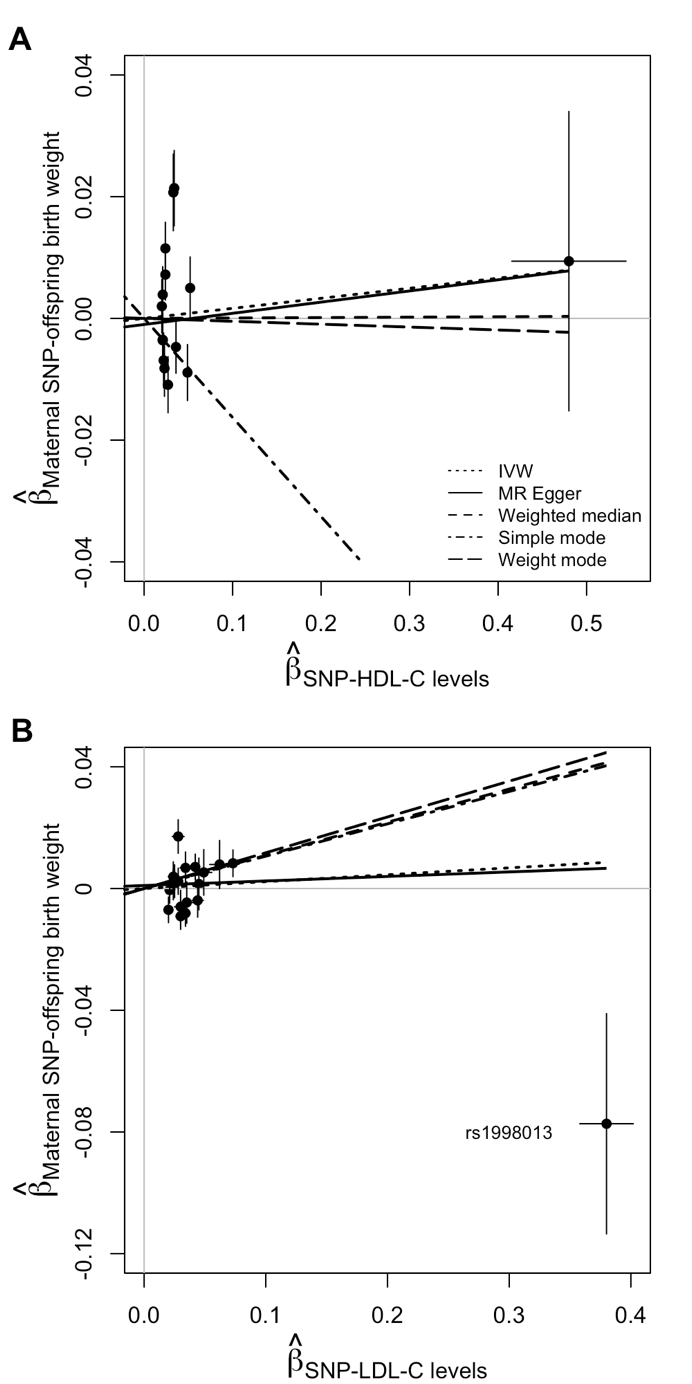
Supplementary Figure 2. Scatter plots of the estimated SNP effects on offspring birth weight plotted against the estimated SNPs effects on the maternal (A) HDL-C, (B) LDL-C levels using SNPs from the “Restricted Set”.** The slopes of the lines are the estimated causal effects of the maternal lipid levels on offspring birth weight, estimated using different MR methods (i.e. inverse variance weighted, MR Egger regression, weighted median, simple mode-based, and weighted mode-based). An outlier SNP, rs1998013, is labelled in (B). It is distal from all other LDL-C associated SNPs and drives the causal effect toward the null.


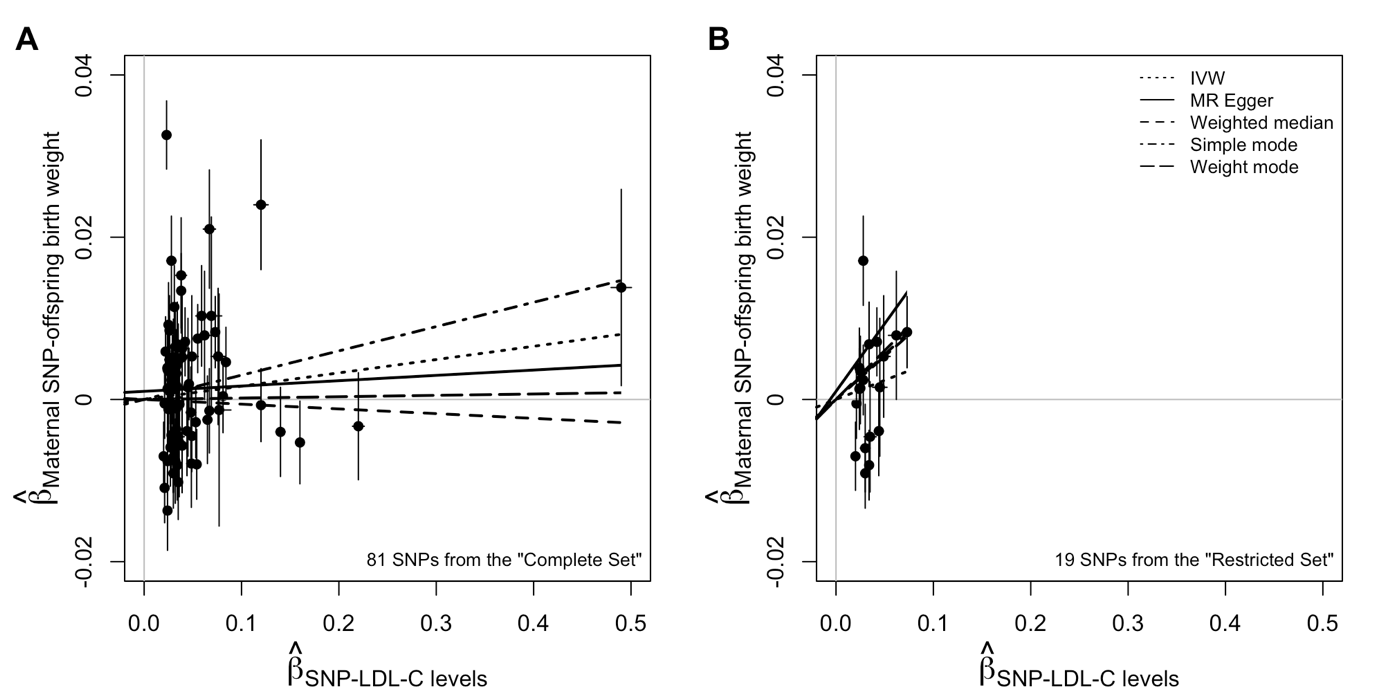


**Supplementary Figure 3. Scatter plots of the estimated SNP effects on offspring birth weight plotted against the estimated SNP effects on maternal LDL-C level using (A) 81 SNPs from the “Complete Set” and (B) 19 SNPs from the “Restricted Set” with the outlier SNP rs1998013 removed.** Slopes of lines are the causal effects of the maternal lipid levels on offspring birth weight estimated using different MR models.
